# A Novel Bayes Approach to Impervious Surface Extraction from High-Resolution Remote Sensing Images

**DOI:** 10.3390/s22103924

**Published:** 2022-05-22

**Authors:** Mingchang Wang, Wen Ding, Fengyan Wang, Yulian Song, Xueye Chen, Ziwei Liu

**Affiliations:** 1 College of Geo-Exploration Science and Technology, Jilin University, Changchun 130026, China; wangmc@jlu.edu.cn (M.W.); wangfy@jlu.edu.cn (F.W.); ylsong19@mails.jlu.edu.cn (Y.S.); lzw21@mails.jlu.edu.cn (Z.L.); 2 Key Laboratory of Urban Land Resources Monitoring and Simulation, Ministry of Natural Resources, Shenzhen 518000, China; xueye31@163.com

**Keywords:** impervious surface, Bayes discriminant analysis, Gaussian prior model, GF-2, Sentinel-2

## Abstract

Impervious surface as an evaluation indicator of urbanization is crucial for urban planning and management. It is necessary to obtain impervious surface information with high accuracy and resolution to meet dynamic monitoring under rapid urban development. At present, the methods of impervious surface extraction are primarily based on medium-low-resolution images. Therefore, it is of theoretical and application value to construct an impervious surface extraction method that applies to high-resolution satellite images and can solve the shadow misclassification problem. This paper builds an impervious surface extraction model by Bayes discriminant analysis (BDA). The Gaussian prior model is incorporated into the Bayes discriminant analysis to establish a new impervious surface extraction model (GBDA) applicable to high-resolution remote sensing images. Using GF-2 and Sentinel-2 remote sensing images as experimental data, we discuss and analyze the applicability of BDA and GBDA in impervious surface extraction of high-resolution remote sensing images. The results showed that the four methods, SVM, RF, BDA and GBDA, had OA values of 91.26%, 94.91%, 94.64% and 97.84% and Kappa values of 0.825, 0.898, 0.893 and 0.957, respectively, in the extraction results of GF-2. In the results of effective Sentinel-2 extraction, the OA values of the four methods were 87.94%, 91.79%, 92.19% and 93.51% and the Kappa values were 0.759, 0.836, 0.844 and 0.870, respectively. Compared with the support vector machine (SVM), random forest (RF) and BDA methods, GBDA has significantly improved the extraction accuracy. GBDA enhances the robustness and generalization ability of the model and can improve the shadow misclassification phenomenon of high-resolution images. The model constructed in this paper is highly reliable for extracting impervious surfaces from high-resolution remote sensing images, exploring the application value of Bayes discriminant analysis in impervious surface extraction and providing technical support for impervious surface information of high spatial resolution and high quality.

## 1. Introduction

Impervious surfaces are surfaces covered by various impervious materials, such as roofs, roads, squares and parking lots made of tiles, asphalt, cement, concrete, etc. [1,2]. The impervious surface can express urban land use and cover and has an essential impact on urban climate and temperature and is also a vital evaluation indicator of the degree of urbanization and urban environmental quality [3,4,5]. Impervious surface information provides the necessary data for urban planning, resource and environmental management and the construction of ecological civilization [6]. Obtaining fast and accurate information on urban impervious surfaces on a regional and global scale is essential for urban management and future planning decisions [7,8,9,10].

Currently, remote sensing technology is an effective tool for obtaining impervious surface information [11]. Remote sensing technology can provide accurate spatial and temporal information on the Earth’s surface by allowing simultaneous observation of large areas in a short period [12]. With the development of satellite images in recent years, various low-, medium- and high-resolution satellite images have been widely used for impervious surface extraction studies [13]. However, in the current research, to quickly obtain information on large impervious surfaces, medium-resolution remote sensing images are still used as the primary research scale, especially Landsat series data [14,15]. With the rapid development of cities, impervious surface information with high spatial resolution and localized details are equally essential [16]. High-resolution imagery as a material for extracting impervious surfaces can significantly reduce the mixed pixel problem and reflect more detailed land cover characteristics [17,18]. According to the definition of impervious surface resolution level, high resolution refers to the spatial resolution of fewer than 10 m [19]. GF-2 and Sentinel-2 data are of high research value for extracting high-resolution impervious surface information [20,21]. Sentinel-2 has 10 m resolution multispectral imagery [22], allowing for clearer impervious surface boundary identification than 30 m Landsat imagery [23]. GF-2 is one of the sub-meter high-resolution remote sensing data sources that can provide material for obtaining detailed information on local impervious surfaces [16]. However, there are shadow problems in extracting impervious surfaces using high-resolution images [24]. These shadows are often caused by high-rise buildings, which affect the accuracy of impervious surface detection [25]. Therefore, various methods for extracting impervious surfaces of remote sensing images have also been developed and utilized, mainly including spectral mixture analysis (SMA) [26,27], remote sensing inversion index-based methods [15] and classification-based methods [28,29,30]. SMA is a method to solve the problem of mixed pixels. SMA is mainly applied to the impervious surface information extraction of medium-resolution remote sensing images [31,32]. However, the SMA method has high computational complexity [33,34]. The use of the SMA method is limited when the remote sensing data band is less or the resolution is high [1]. Remote sensing-based impervious surface inversion indexes are suitable for large impervious surface extraction [35]. At this stage, many remote sensing inversion indices [36,37,38,39] have been developed and used to enhance impervious surface information [40,41]. However, the index method is often used to extract impervious surface information from medium-resolution images and lacks the exploration of high-spatial-resolution images [42]. Among classification-based methods, unsupervised classification algorithms are rarely applied to urban impervious surface extraction because of their low classification accuracy [25]. Supervised classification can separate impervious surfaces from spectrally similar pervious surfaces. Many global and regional land cover products that can be used for impervious surface information analysis have been released in recent years through a supervised classification framework [43,44]. However, supervised classification produces products with low spatial resolution and limited local accuracy [12]. In summary, medium-low-resolution impervious surface mapping techniques have matured, but relatively few studies have been conducted on high-resolution impervious surface information extraction [16]. To meet the current demand for the rapid and accurate acquisition of high-resolution and high-accuracy impervious surface information under urbanization, the impervious surface extraction method, which applies to high-spatial-resolution satellite images, can solve shadow misclassification and has a simple and effective extraction process, which is of high research value [15].

Discriminant analysis is a multivariate statistical method that objectively uses multiple factors to classify predictive objects [44]. Bayes discriminant constructs a multivariate discriminant model based on the Bayes criterion to discriminate against the predicted object [45], which is a method to find the best division under the condition of optimizing the minimum false loss [46,47]. Bayes discriminant analysis has been applied to many fields and produces more accurate predictions, especially risk prediction [48,49,50], disease prediction [51,52] and chemometric statistical analysis [47,53,54]. However, studies applying Bayes discriminant analysis to urban impervious surface extraction are rare.

At present, how to effectively and quickly obtain high-accuracy impervious surface information from high-resolution remote sensing images and reduce the misclassification phenomenon generated by shadows are urgent problems to be solved. Introducing prior knowledge can improve extraction accuracy and reduce shadow misclassification. To this end, this paper constructs an impervious surface extraction model based on Bayes discriminant analysis and explores the application of Bayes discriminant analysis in impervious surface information extraction. We improve the prior of the BDA model to the Gaussian prior and propose an impervious surface extraction model (GBDA) applicable to high-spatial- resolution remote sensing images. Impervious surface information extraction experiments on GF-2 and Sentinel-2 remote sensing images were conducted to verify the performance and applicability of the GBDA model on multi-scale high-resolution images. The main contributions to this paper are as follows:

(1) This paper proposes a Gaussian prior-based Bayes discriminant analysis impervious surface extraction model that can extract highly accurate impervious surface information and clear boundaries;
(2) The impervious surface model constructed based on Bayes discriminant analysis has the advantages of simple process, high computational efficiency and good comprehensive performance and can be used to extract impervious surface information from multi-scale high-resolution remote sensing images. It avoids the waste of computational resources, reduces the influence of subjective factors brought by sample selection in the extraction process and improves extraction accuracy;
(3) The multivariate Gaussian distribution model is used to construct the prior model because of its wide adaptability and advantage in analyzing complex statistics. The GBDA model incorporating Gaussian prior enhances the generalization ability and improves the robustness, effectively improving the extraction accuracy of impervious surfaces of high-resolution remote sensing images and reducing the shadow misclassification phenomenon.

## 2. Methodology

**Figure 1:**
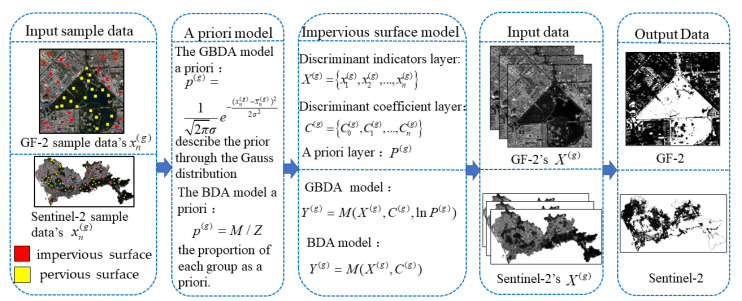
Overview of the GBDA and BDA.

The method of extracting impervious surfaces based on GBDA and BDA consists of three parts: (1) Constructing a priori models: The a priori models of GBDA and BDA are constructed based on the discriminant values $x_{n}^{\left(g\right)}$ of the training samples, the total number of samples Z and the sample size M of the group $A^{\left(g\right)}$, respectively. (2) Calculate the discriminant coefficient and construct the impervious surface extraction model: According to Equations (8)–(12), the discriminant layer $C^{\left(g\right)}$ is obtained from the discriminant index value $x_{n}^{\left(g\right)}$ of the training samples. Then the GBDA and BDA models can be constructed by combining the obtained a priori models. (3) Impervious surface extraction: Various image features are extracted as discriminant indicator layer $X^{\left(g\right)}$. $X^{\left(g\right)}$ is used as input data for the model. Output $Y^{\left(g\right)}$ value follows the discriminative principle that the larger the obtained $Y^{\left(g\right)}$ value is, the more likely the pixel belongs to the group $A^{\left(g\right)}$ and the final impervious surface extraction result is obtained. The overall structure of the algorithm in this paper is shown in Figure 1.

### 2.1. A Novel Bayes Method for Impervious Surface Extraction from Remote Sensing Images

The impervious surface extraction model for Bayes discriminant analysis is constructed based on the Bayes criterion. The Bayes criterion is to find an optimal division under the principle of minimizing the average loss of misclassification in the division [55]. Specifically, let there be a total of G(G=2) categories and the samples are divided into g categories (g=1,2), which are noted as group $A^{\left(1\right)}$ impervious surface, group $A^{\left(2\right)}$ pervious surface and have N discriminative indicators. The Bayes criterion can be expressed as the maximum of the product of the prior and the probability density function. Specifically, $p^{\left(g\right)}$ denotes the prior, $f^{\left(g\right)}(x)$ is the probability density corresponding to the gth category and the Bayes criterion can be expressed as the maximum value of $p^{\left(g\right)}f^{\left(g\right)}(x)$. Then, Bayes discriminant analysis for impervious surface extraction is equivalent to deriving a quantity similar to the maximum posterior probability of each group, i.e.,

(1) $$Y^{\left(g\right)}=p^{\left(g\right)}f^{\left(g\right)}(x)$$

From the perspective of the sample multivariate distribution, Equation (1) is shown as:

(2) $$Y^{\left(g\right)}=p^{\left(g\right)}f^{\left(g\right)}(x)=C_{0}^{\left(g\right)}+C_{1}^{\left(g\right)}x_{1}^{\left(g\right)}+C_{2}^{\left(g\right)}x_{2}^{\left(g\right)}+\ldots +C_{n}^{\left(g\right)}x_{n}^{\left(g\right)}+\ln P^{\left(g\right)}$$

The value of $Y^{\left(g\right)}$ reflects the likelihood of the pixel appearing in category g. The larger the $Y^{\left(g\right)}$, the more likely the pixel to be judged is to appear in category g.

#### 2.1.1. The Prior Model

Before constructing the impervious surface extraction model, the prior model $p^{\left(g\right)}$ must be built. We built two prior models. One was built simply by using the proportion of each group as the prior models, i.e.,

(3) $$p^{\left(g\right)}=M/Z$$

where Z is the total number of pixels in the sample and M is the number of pixels in $A^{\left(g\right)}$.

The other is model is the Gaussian prior. The multivariate Gaussian distribution model is essential in multivariate analysis because of its wide adaptability and advantage in analyzing complex statistics [56]. Therefore, we describe the prior through the Gaussian distribution so that the prior distribution of the group $A^{\left(g\right)}$ follows a Gaussian distribution with mean ${\bar{x}}_{n}^{\left(g\right)}$:

(4) $$p^{\left(g\right)}=\frac{1}{\sqrt{2\pi }\sigma }e^{-\frac{{\left(x_{n}^{\left(g\right)}-{\bar{x}}_{n}^{\left(g\right)}\right)}^{2}}{2\sigma^{2}}}$$

where $x_{n}^{\left(g\right)}$ is the value of the nth discriminant in the group $A^{\left(g\right)}$, ${\bar{x}}_{n}^{\left(g\right)}$ is the sample mean of the nth feature of category g:

(5) $${\bar{x}}_{n}^{\left(g\right)}=\frac{1}{M}\sum_{m=1}^{M}x_{mn}^{\left(g\right)}(g=1,2;n=1,2,\ldots ,N)$$

(6) $${\bar{x}}_{}^{\left(g\right)}=({\bar{x}}_{1}^{\left(g\right)},{\bar{x}}_{2}^{\left(g\right)},\ldots ,{\bar{x}}_{N}^{\left(g\right)})$$

#### 2.1.2. Gaussian Prior-Based Bayes Discriminant Analysis Impervious Surface Extraction Model

The Gaussian prior-based Bayes discriminant analysis impervious surface extraction model is obtained from Equations (2) and (4). It consists of the discriminant indicator layer $X^{\left(g\right)}$, discriminant coefficient layer $C^{\left(g\right)}$ and the prior layer $P^{\left(g\right)}$:

(7) $$Y^{\left(g\right)}=M(X^{\left(g\right)},C^{\left(g\right)},\ln P^{\left(g\right)})$$

Discriminant indicator layer $X^{\left(g\right)}=\left\{x_{1}^{\left(g\right)},x_{2}^{\left(g\right)},\ldots ,x_{n}^{\left(g\right)}\right\}$: $x_{n}^{\left(g\right)}$ is the value of the nth discriminant indicator in group $A^{\left(g\right)}$. This paper chooses the spectral bands of remote sensing images as the discriminant indicators. $x_{n}^{\left(g\right)}$ is the grayscale value of each band image.

Discriminant coefficient layer $C^{\left(g\right)}=\left\{C_{0}^{\left(g\right)},C_{1}^{\left(g\right)},\ldots ,C_{n}^{\left(g\right)}\right\}$: $C^{\left(g\right)}$ is the coefficient of the discriminant indicators in the discriminant model, where $C_{0}^{\left(g\right)}$ is a constant term:

(8) $$(C_{1}^{\left(g\right)},C_{2}^{\left(g\right)},\ldots ,C_{N}^{\left(g\right)})=(Z-G)S^{-1}{\bar{x}}^{(g)\prime }$$

(9) $$C_{0}^{\left(g\right)}=-\frac{1}{2}\sum_{n=1}^{N}C_{n}^{\left(g\right)}x_{n}^{\left(g\right)}$$

where Z is the total number of samples, M is the number of samples in the category g, ${\bar{x}}_{n}^{\left(g\right)}$ is the sample mean of the nth (n=1,2,…,N) feature of the gth category and ${\bar{x}}^{(g)\prime }$ is the transpose of the vector consisting of the characteristic mean.

$S^{-1}$ is the inverse matrix of the inter-factor correlation number matrix S. The inter-factor correlation number matrix S:

(10) $$S=\left[\begin{array}{l} S_{11},S_{12},\ldots ,S_{1N}\\ S_{21},S_{21},\ldots ,S_{2N}\\ \ldots \\ S_{N1},S_{N2},\ldots ,S_{NN} \end{array}\right]$$

(11) $$S_{kl}=\sum_{g=1}^{G}S_{kl}^{\left(g\right)}=S_{kl}^{\left(1\right)}+S_{kl}^{\left(2\right)}+\ldots +S_{kl}^{\left(G\right)}\;(k=1,2,\ldots ,N;l=1,2,\ldots ,N)$$

(12) $$S_{kl}^{\left(g\right)}=S_{lk}^{\left(g\right)}=\sum_{i=1}^{M}\left(x_{ki}^{\left(g\right)}-{\bar{x}}_{k}^{\left(g\right)})(x_{li}^{\left(g\right)}-{\bar{x}}_{l}^{\left(g\right)}\right)$$

where $x_{ki}^{\left(g\right)}$ is the value of the kth feature of the ith sample in the gth category and the value of $x_{li}^{\left(g\right)}$ is the same.

The prior layer $P^{\left(g\right)}$: The prior model (4) constructed by Gaussian distribution is incorporated into the impervious surface extraction model. The prior model $P^{\left(g\right)}$ in GBDA exists in the form of $\ln P^{\left(g\right)}$, which can be transformed into the following format:

(13) $$\ln p^{\left(g\right)}=\lambda^{\left(g\right)}{\left\|x^{\left(g\right)}-{\bar{x}}^{\left(g\right)}\right\|}_{2}^{2}$$

where $\lambda^{\left(g\right)}$ is the regularization parameter.

Since $p^{\left(g\right)}$ in (3) is a constant value, the impervious surface extraction model based on Bayes discriminant analysis can be obtained from (2) and (3), which consist of the discriminant indicator layer $X^{\left(g\right)}$ and discriminant coefficient layer $C^{\left(g\right)}$:

(14) $$Y^{\left(g\right)}=M(X^{\left(g\right)},C^{\left(g\right)})$$

### 2.2. Extracting Features and Collecting Training Samples

**Figure 2:**
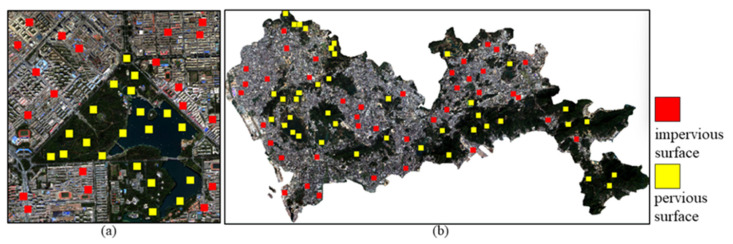
Training sample dataset labeling diagram: (**a**) GF-2 image samples; (**b**) Sentinel-2 image samples.

We chose three bands in the wavelength range of 0.45–0.51 μm, 0.53–0.59 μm and 0.64–0.67 μm as discriminative indicators. The training sample dataset of GF-2 is marked in Figure 2a, with 204,020 impervious surface samples and 204,020 pervious surface samples in the sample dataset. The training sample dataset of Sentinel-2 is shown in Figure 2b, with 408,040 impervious surface and pervious surface samples each. To verify the effectiveness of the method in this paper, we randomly select different numbers of samples from the existing sample set to train the model in this paper.

### 2.3. Accuracy

To comprehensively evaluate the impervious surface extraction model, we used precision, recall, $F_{1}$ value, Overall Accuracy (OA) and Kappa coefficient for accuracy evaluation [57,58]. Precision is the ratio of samples that are actually impervious among all samples predicted to be impervious and recall is the ratio of samples that are predicted to be impervious among those that are actually impervious. $F_{1}$ value is a statistical measure of the accuracy of a binary classification model, which considers both the precision and recall and is a reconciled average of the model’s precision and recall. The characteristics of different models in impervious surface extraction can be better analyzed based on precision and recall. However, the calculation results of precision, recall and $F_{1}$ value excessively depend on the number of samples and categories and there will be uncertainties. Combining the OA value and Kappa coefficient can evaluate the accuracy of the results more objectively and fairly. We calculated OA, Kappa, precision and recall based on confusion matrix and calculated $F_{1}$ value based on precision and recall.

## 3. Experiments and Results

### 3.1. Experimental Areas and Data

#### 3.1.1. Experimental Areas

**Figure 3:**
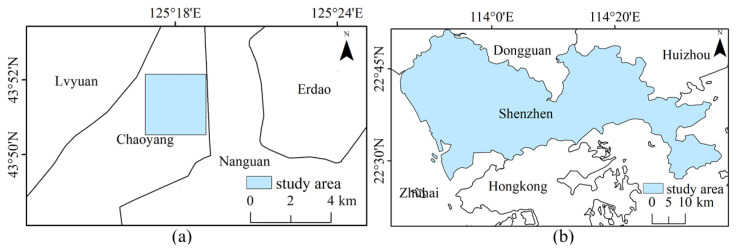
Experimental areas: (**a**) Chaoyang District, Changchun City; (**b**) Shenzhen City.

Changchun is located in the geographical center of Northeast China. Changchun is at a medium level of development, but the city has experienced rapid economic growth and significant urbanization in recent years. Chaoyang District is located in the south-central part of Changchun’s central city, which is a representative area of Changchun’s urbanization process. There are certain research implications of using the Chaoyang District as a research object for urban development. This paper selects some areas within Chaoyang District as the study area (Figure 3a).

Shenzhen is located in the south of Guangdong Province, with nine administrative districts and one new district under its jurisdiction. With the rapid development of urbanization and the rapid expansion of impervious surfaces, the land cover composition of Shenzhen presents a high degree of heterogeneity, which has great potential in the field of urban remote sensing research (Figure 3b).

#### 3.1.2. Remote Sensing Data

**Figure 4:**
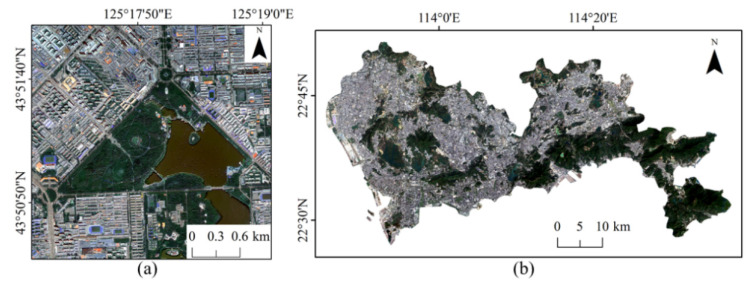
Image Data: (**a**) GF-2; (**b**) Sentinel-2.

Gaofen-2 (GF-2), one of China’s new generation satellites, was officially put into use in 2015, featuring high spatial resolution and high positioning accuracy, providing data for the production of high-quality remote sensing products [13]. GF-2 has two types of images: one is a multispectral image covering four spectral bands in the near-infrared range with a spatial resolution of 4 m; the other is a panchromatic image with a spatial resolution of 1 m in the visible spectrum [59]. This paper selected the GF-2 image of the study area in Chaoyang District for the experiment. We decided on images with no cloud coverage. Since a single scene covered the study region, image mosaicking was not considered. The multispectral image was fused with the panchromatic image by the NNDiffuse Pan Sharpening method into a 1 m resolution image with an image size of 3000 × 3000 pixels (Figure 4a).

The Sentinel-2A satellite carries the Multi-Spectral Imager (MSI), covering 13 spectral bands. The spatial resolution of the near-infrared, red, green and blue bands is 10 m and the resolution of the red-edge band and the two short-wave infrared bands of the 11th and 12th is 20 m. The resolution of the coastal/aerosol band, the water vapor band and the short-wave infrared band in the 10th band is 60 m. Sentinel-2 [22] images can provide clearer impervious surface boundary identification and are commonly used to extract impervious surface information [23]. We selected the Sentinel-2 image of the Shenzhen study area for the experiment. There are five scenes of Sentinel-2 remote sensing images covering the study area and the images are cloud-free and of good quality. The cropped images were mosaicked into one image containing the study area and the Sentinel-2 image of the study area is shown in Figure 4b.

### 3.2. Impervious Surface Extraction Experiments Based on GF-2 Images

The data used are GF-2 remote sensing images of an area in Chaoyang District (Figure 4a) with 1 m resolution, containing three wavelength bands in the range of 0.45–0.51 μm, 0.53–0.59 μm and 0.64–0.67 μm and an image size of 3000 × 3000 pixels. Four different numbers of samples were selected from the existing samples (Figure 2a) to train the model in this paper to verify the effectiveness of our model. The four groups of samples had the same number of impervious and pervious surfaces: 40,000, 60,000, 80,000 and 100,000, respectively. We used MATLAB software to write programs to implement the methods in this paper. The SVM selected the radial basis function to train the classifier and the number of RF trees was 100. The OA, Kappa coefficient and $F_{1}$ value of the extraction results of the four methods were calculated. A total of 27,000 test samples were randomly created (13,500 each for impervious and pervious surfaces) and the accuracy results are shown in Table 1.

**Figure 5:**
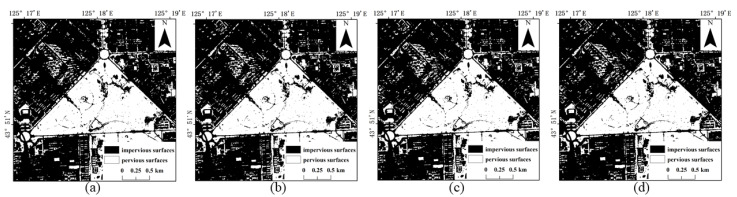
Results of impervious surface extraction with SVM: (**a**) 40,000 samples; (**b**) 60,000 samples; (**c**) 80,000 samples; (**d**) 100,000 samples.

**Figure 6:**
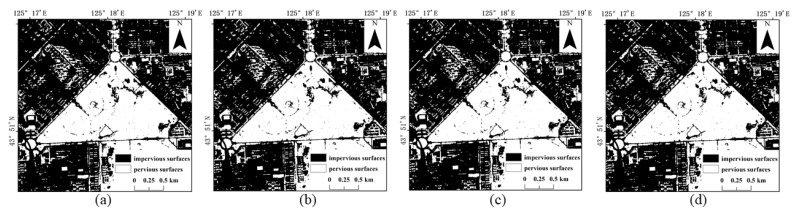
Results of impervious surface extraction with RF: (**a**) 40,000 samples; (**b**) 60,000 samples; (**c**) 80,000 samples; (**d**) 100,000 samples.

**Figure 7:**
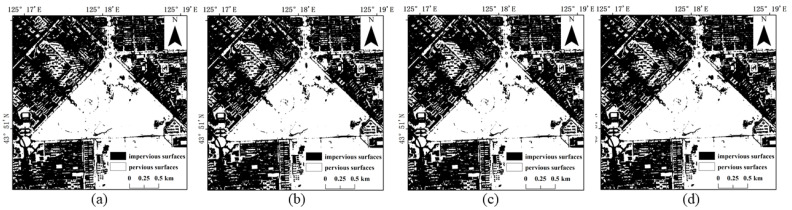
Results of impervious surface extraction with BDA: (**a**) 40,000 samples; (**b**) 60,000 samples; (**c**) 80,000 samples; (**d**) 100,000 samples.

**Figure 8:**
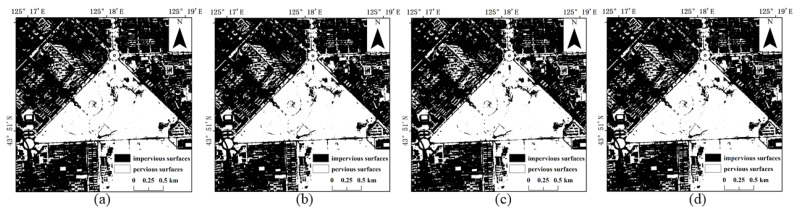
Results of impervious surface extraction with GBDA: (**a**) 40,000 samples; (**b**) 60,000 samples; (**c**) 80,000 samples; (**d**) 100,000 samples.

The results of the four methods using different numbers of training samples to extract impervious surfaces are shown in Figure 5, Figure 6, Figure 7 and Figure 8. Comparison with the actual image (Figure 4a) reveals that the results obtained by SVM and RF produce more misclassification of the pervious surface into the impervious surface, with the misclassification of SVM being more serious. The results of the BDA method are exactly the opposite of the two, producing more impervious surfaces with omission classification. Compared with the three, the extraction accuracy of the GBDA method after optimizing the prior was significantly improved, effectively balancing the occurrence of the above two types of misclassification problems so that impervious and pervious surfaces can be correctly identified.

Among the four methods, SVM obtained the lowest accuracy and the two methods, BDA and RF, had similar extraction accuracy with higher accuracy values than SVM. Compared to SVM, the maximum increase in OA of BDA was 3.77%, the maximum increase in Kappa value was 0.0752 and the maximum increase in $F_{1}$ value was 0.0276. The OA and Kappa of GBDA with a prior optimization were substantially improved compared with the other three methods. Compared to BDA, GBDA had a maximum increase of 3.23% in OA, 0.0647 in Kappa and 0.0352 in $F_{1}$ value.

### 3.3. Impervious Surface Extraction Experiments Based on Sentinel-2 Images

The data were selected from Sentinel-2 remote sensing images of Shenzhen, Guangdong Province (Figure 4b), with a resolution of 10 m, containing three wavelength bands in the range of 0.45–0.51 μm, 0.53–0.59 μm and 0.64–0.67 μm. Four different numbers of randomly selected samples from the existing impervious surface and pervious surface samples were trained for the four classifiers, respectively, to verify the method’s effectiveness in Sentinel-2 images in this paper and to record the accuracy of the impervious surface extraction results. The four groups of samples had the same number of impervious and pervious surfaces: 80,000, 120,000, 160,000 and 200,000, respectively. We still used MATLAB software to write programs to implement the methods in this paper. The RBF radial basis function was still chosen to train the SVM classifier with the random forest trees of 100.

**Figure 9:**
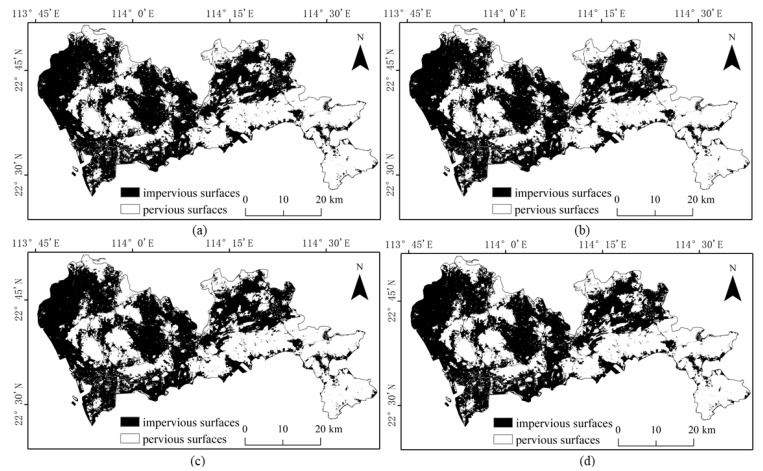
Results of impervious surface extraction with SVM: (**a**) 80,000 samples; (**b**) 120,000 samples; (**c**) 160,000 samples; (**d**) 200,000 samples.

**Figure 10:**
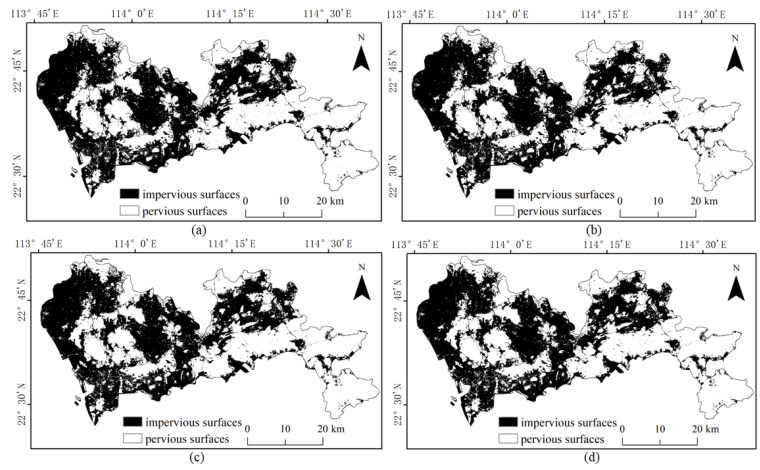
Results of impervious surface extraction with RF: (**a**) 80,000 samples; (**b**) 120,000 samples; (**c**) 160,000 samples; (**d**) 200,000 samples.

**Figure 11:**
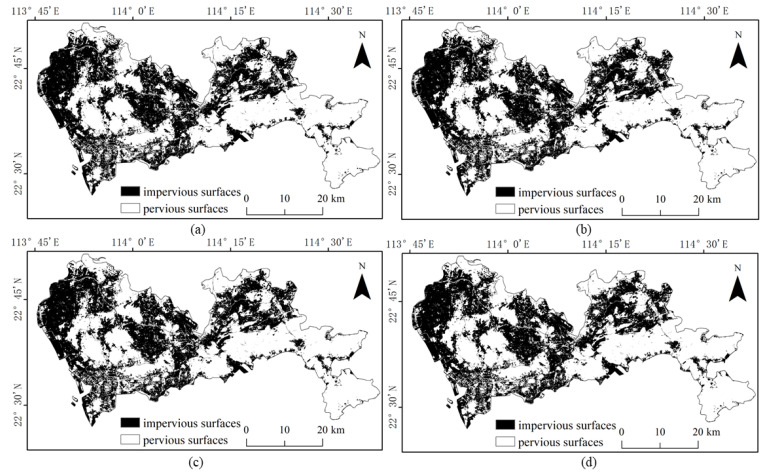
Results of impervious surface extraction with BDA: (**a**) 80,000 samples; (**b**) 120,000 samples; (**c**) 160,000 samples; (**d**) 200,000 samples.

**Figure 12:**
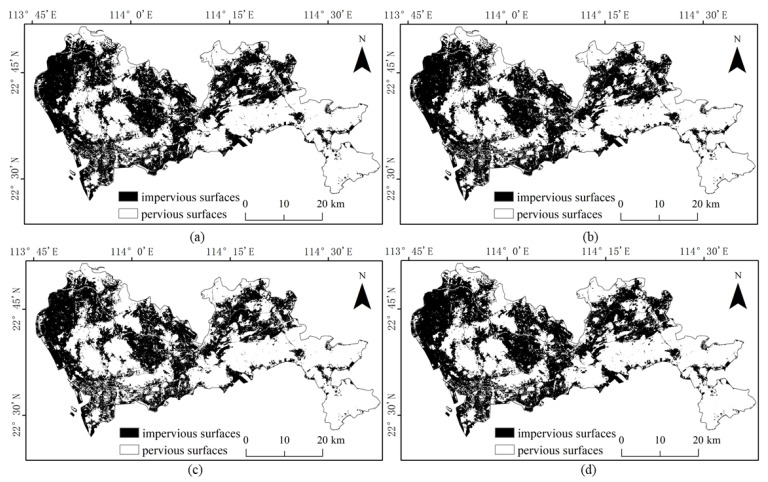
Results of impervious surface extraction with GBDA: (**a**) 80,000 samples; (**b**) 120,000 samples; (**c**) 160,000 samples; (**d**) 200,000 samples.

Referring to the Shenzhen Sentinel-2 remote sensing image, it was found by visual inspection that SVM (Figure 9) and RF (Figure 10) were prone to classifying water and bare ground as impervious surfaces. Compared with SVM, RF had relatively minor misclassifications for these two categories. The BDA (Figure 11) method produced better classification results for bare soils and meandering rivers with similar spectral characteristics to impervious surfaces. However, BDA was prone to shadow misclassification due to the similarity between house shadows and certain spectral features of pervious surfaces. The comparison of the results revealed that the GBDA (Figure 12) method, which optimizes a prior, can effectively reduce the shadow misclassification phenomenon.

A total of 200,000 test samples were randomly created (100,000 each for impervious and pervious surfaces) and the accuracy results are shown in Table 2. The accuracy of this experiment achieved the same pattern as Experiment 1: SVM had the lowest accuracy among the four methods; RF and BDA were next, both had similar accuracy values and GBDA had the highest accuracy. GBDA showed a maximum increase of 7.96% in OA, 0.1592 in Kappa and 0.0638 in $F_{1}$ value compared to SVM. GBDA showed a maximum increase of 1.39% in OA, 0.0278 in Kappa and 0.0168 in $F_{1}$ value compared to BDA.

### 3.4. The Analysis of Precision and Recall

**Figure 13:**
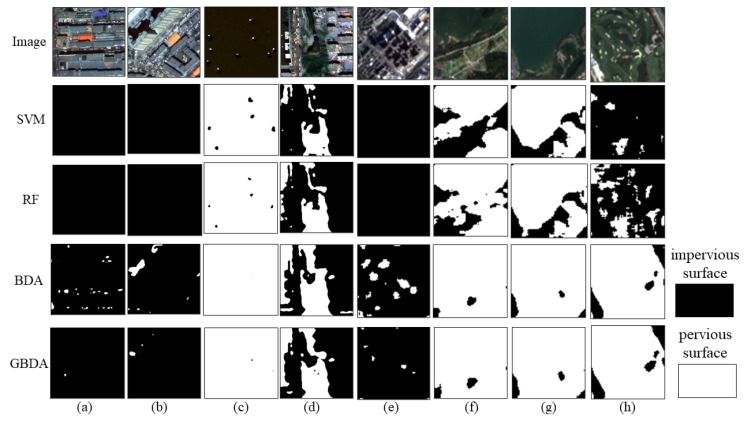
Local view of GF-2 and Sentinel-2: (**a**) GF-2 shaded 1; (**b**) GF-2 shaded 2; (**c**) GF-2 water surface; (**d**) GF-2 woodland and water; (**e**) Sentinel-2 shading; (**f**) Sentinel-2 bare soil; (**g**) Sentinel-2 water surface 1; (**h**) Sentinel-2 water surface 2.

The precision and recall of the GF-2 and Sentinel-2 image impervious surface extraction results were obtained based on the test samples (Table 3 and Table 4). Precision is the number of samples that are actually impervious among all samples predicted to be impervious and recall is the number of samples that are predicted to be impervious among those that are actually impervious. The characteristics of different models in impervious surface extraction can be better analyzed based on precision and recall. Additionally, some partial parts are selected to show the details (Figure 13) to better demonstrate the extraction results.

In the precision and recall results of GF-2 (Table 3), the recall of SVM and RF was higher than the precision. The recall of both reached 99%. However, RF had higher precision, with a maximum improvement of 6.13% than SVM. From Figure 13c,d, it can be seen that SVM and RF can easily misclassify the pervious surface as an impervious surface. The BDA results were the opposite of both, with a precision of 99% and a recall of about 89%. Figure 13a,b shows an omission classification of building shadows by BDA. GBDA reconciled the precision and recall of the first three methods. GBDA improved the precision compared to RF with a maximum improvement of 6.98% and the recall compared to BDA with a maximum improvement of 8.50%.

The precision and recall results of Sentinel-2 (Table 4) show the same for SVM and RF with high recall and low precision and BDA with high precision and low recall. From Figure 13f–h, SVM and RF were more likely to classify bare soil and water as impervious surfaces and Figure 13e shows that BDA still produced an omission classification for building shadows. The maximum improvement in GBDA precision was 4.96% compared to RF and the maximum improvement in GBDA recall was 5.68% compared to BDA.

## 4. Discussion

### 4.1. The Role of the Prior Model Optimization

The BDA model only consists of the discriminant indicator and discriminant coefficient layers and lacks the prior layer compared with GBDA. According to the precision and recall of the BDA model for extracting impervious surfaces (Table 3 and Table 4), it is known that BDA has a low recall and high precision. It is also easy to see from the extracted local view (Figure 13) that the BDA extraction results produce the phenomenon of leaving out the shadows of the buildings. We believe that this is inextricably linked to the lack of an a priori layer in the BDA model. The BDA model is constructed under the Bayes criterion. The Bayes criterion is to find an optimal division under the principle of minimizing the loss of impervious surface misclassification in the division. The smaller the loss value can make the discriminative accuracy higher, conducive to the extraction of impervious information. However, this criterion also omits target categories due to the avoidance of misclassification losses. Therefore, we can judge that the Bayes criterion plays a decisive role in the BDA model and the advantage of the prior model is not shown.

The multivariate Gaussian distribution model is vital in multivariate analysis because of its wide adaptability and the advantage of analyzing complex statistics. Therefore, we first constructed the prior model using Gaussian distribution and then integrated the Gaussian prior model into Bayes discriminant analysis to build the GBDA model. From the extraction results and accuracy of GBDA, compared with BDA, GBDA effectively suppresses the phenomenon of building shadow omission while improving the extraction accuracy. It is experimentally demonstrated that the optimization of the Gaussian prior model enhances the robustness and generalization ability of the impervious surface model.

### 4.2. Feasibility and Superiority of GBDA Model in Extracting Impervious Surface

In this paper, the construction of a high-resolution image impervious surface extraction model of GBDA based on Bayes discriminant analysis depends on three main points. First, Bayes discriminant analysis considers the prior knowledge of impervious surfaces. Second, the discriminant process obeys the minimum misjudgment loss. Third, the high-resolution image mitigates the mixed-image phenomenon and it fits better with the GBDA model.

A basic assumption of Bayes discriminant analysis is that there is some knowledge of the object under study before discriminating and this knowledge is usually described prior. Such a prior can improve the possibility of accurate discrimination, but commonly used impervious surface extraction methods typically do not incorporate prior knowledge. Through experiments, we confirmed the reliability of using a prior.

Misclassification loss refers to the loss that would result from misclassifying samples belonging to one class as other classes. Generally, misclassification losses are compared rather than quantified, but such losses can be quantified using Bayes discriminant analysis. According to Bayes criterion, the maximum value of $p^{\left(g\right)}f^{\left(g\right)}(x)$ is equivalent to the maximum posterior probability and the maximum posterior probability is equivalent to the minimum misclassification loss. Therefore, the maximum amount of $Y^{\left(g\right)}$ we found by the model is equivalent to the minimum average misclassification loss. The smaller the loss value is, the higher the discriminative accuracy. However, this method of quantifying misclassification losses is also lacking in other discriminant analysis methods and impervious surface extraction methods.

Bayes discriminant analysis performs impervious surface extraction by discriminating each pixel. The impervious surface in the actual ground consists of a combination of different materials, each of which has its own characteristics. The problem of mixed pixels in medium- and low-resolution images is serious, weakening the model robustness when constructing the model and raising the difficulty of discriminating unknown pixels. Using high-resolution images to extract impervious surfaces can significantly reduce the mixed pixel problem, matching the extraction process of GBDA for impervious surfaces. Therefore, due to the uniqueness and superiority of Bayes discriminant analysis in the above three aspects, this paper selected Bayes discriminant analysis to construct a high-resolution remote sensing image impervious surface extraction model and verified the superiority of the model through experiments.

### 4.3. Uncertainties and Limitations

Although the accuracy evaluation shows the excellent performance of the method in this paper, there are uncertainties and limitations in our approach. The uncertainty in GBDA is expressed in the regularization parameter $\lambda^{\left(g\right)}$ of the prior model. However, the search for the optimal $\lambda^{\left(g\right)}$-value is uncertain. On the one hand, we set a series of $\lambda^{\left(g\right)}$-values for the GBDA method to find the most suitable one. On the other hand, the optimal parameter values vary for different satellite images and different numbers of training sample sets of the same image. Although GBDA produces better extraction results, how to find the best parameter quickly is an urgent problem we need to solve.

High-resolution imagery can provide more delicate impervious surface information. Still, it should also ensure that higher extraction quality and spatial and temporal continuity of impervious information are obtained, which is equally essential for urban planning and development. It is currently more difficult to distinguish different objects with very similar spectral features. In general, the more predictors in the model, the higher the model’s accuracy, but the more complex the calculation. More and more researchers extract various urban elements in remote sensing images by obtaining feature information such as spatio-temporal, texture, color, edge, etc., to obtain more accurate extraction accuracy. The expansion of impervious surfaces is often spontaneous and intentional, especially in rapidly developing areas. In this regard, accurate and efficient monitoring of impervious surfaces’ spatial and temporal dynamics is necessary. Monitoring impervious surface spreading has been difficult because it follows a nonlinear trend of high spatial and temporal heterogeneity. Satellite remote sensing images have unique advantages in such dynamic studies.

Therefore, although the impervious surface extraction model constructed in this paper has the advantages of a simple extraction process and high extraction accuracy, some shortcomings still need to be improved. (1) In this paper, only three bands in the wavelength range of 0.45–0.51 μm, 0.53–0.59 μm and 0.64–0.67 μm are selected as the discriminative indicators of the impervious surface model. The acquisition of other features and data of the image should be further increased, the discriminative indicators should be added and comparative analysis should be performed to find a more suitable combination of features for high-resolution impervious surface information extraction. (2) Data should be available for different periods within the study area and long-term dynamic monitoring of the same area should be conducted to meet better the need for impervious surface information for the urbanization process.

## 5. Conclusions

Impervious surface is an evaluation indicator of urbanization and accurate extraction of impervious surface information is important for urban management and development. The research scales of remote sensing image impervious surfaces mainly focus on low and medium resolutions. There are relatively few studies on the extraction of impervious surface information from high-resolution images. An impervious surface extraction method is of high research value if it applies to high-spatial-resolution satellite images, can solve shadow misclassification and has a simple and effective extraction process to meet the current demand for the rapid and accurate acquisition of high-resolution and high-accuracy impervious surface information under urbanization. This paper proposed a Gaussian prior-based Bayes discriminant analysis impervious surface extraction model and based on the experimental results, this paper draws the following conclusions:

(1) Based on the analysis of the impervious surface extraction results of GF-2 and Sentinel-2 images, both BDA and GBDA methods have achieved better results. It has been proved that using the Bayes discriminant analysis idea to construct an impervious surface extraction model is a suitable method for multi-scale high-resolution remote sensing images with a simple process and high accuracy. Compared with SVM and RF methods, GBDA has better extraction performance;
(2) The BDA uses the percentage of each group value as the prior and the model has fitting problems. In this paper, the prior of BDA is improved to Gaussian prior distribution, which can effectively improve the shadow misclassification phenomenon generated by high-resolution images and improve the extraction accuracy, proving that the improvement of the prior enhances the robustness and generalization ability of the model.

## Figures and Tables

**Table 1:** Accuracy assessment of impervious surfaces of GF-2 remote sensing images.

	SVM			RF			BDA			GBDA		
	OA (%)	Kappa	*F*_1_	OA (%)	Kappa	*F*_1_	OA (%)	Kappa	*F*_1_	OA (%)	Kappa	*F*_1_
40,000	90.87	0.8175	0.9161	94.81	0.8961	0.9505	94.64	0.8927	0.9437	97.84	0.9568	0.9785
60,000	91.06	0.8211	0.9177	94.75	0.8950	0.9500	94.51	0.8901	0.9422	97.74	0.9548	0.9774
80,000	91.26	0.8253	0.9194	94.91	0.8983	0.9514	94.61	0.8921	0.9433	97.78	0.9556	0.9777
100,000	91.07	0.8214	0.9178	94.84	0.8970	0.9563	94.62	0.8924	0.9434	97.82	0.9564	0.9781

**Table 2:** Accuracy assessment of impervious surface of Sentinel-2 remote sensing images.

	SVM			RF			BDA			GBDA		
	OA (%)	Kappa	*F*_1_	OA (%)	Kappa	*F*_1_	OA (%)	Kappa	*F*_1_	OA (%)	Kappa	*F*_1_
80,000	86.89	0.7378	0.8833	91.70	0.8340	0.9223	92.19	0.8438	0.9207	93.51	0.8703	0.9368
120,000	87.94	0.7588	0.8914	91.60	0.8320	0.9215	92.08	0.8415	0.9195	93.46	0.8693	0.9363
160,000	85.50	0.7100	0.8725	91.79	0.8359	0.9231	92.07	0.8415	0.9195	93.46	0.8692	0.9363
200,000	86.92	0.7383	0.8834	91.60	0.8320	0.9214	92.10	0.8420	0.9198	93.44	0.8688	0.9359

**Table 3:** Precision and recall of impervious surfaces of GF-2 remote sensing images.

	SVM		RF		BDA		GBDA	
	Precision (%)	Recall (%)	Precision (%)	Recall (%)	Precision (%)	Recall (%)	Precision (%)	Recall (%)
40,000	84.76	99.67	90.89	99.60	99.30	89.91	97.82	97.87
60,000	85.00	99.70	90.79	99.61	99.38	89.57	97.42	98.07
80,000	85.31	99.69	91.06	99.61	99.39	89.76	97.96	97.59
100,000	85.04	99.68	90.96	99.60	99.35	89.82	97.94	97.69

**Table 4:** Precision and recall of impervious surfaces of Sentinel-2 remote sensing images.

	SVM		RF		BDA		GBDA	
	Precision (%)	Recall (%)	Precision (%)	Recall (%)	Precision (%)	Recall (%)	Precision (%)	Recall (%)
80,000	79.61	99.19	86.64	98.60	93.46	90.72	91.28	96.22
120,000	81.06	99.01	86.53	98.54	93.42	90.53	91.28	96.11
160,000	77.84	99.26	86.81	98.56	93.43	90.52	91.20	96.20
200,000	79.65	99.17	86.52	98.55	93.42	90.58	91.48	95.80

## Data Availability

Not applicable.
